# Multiple pathways promote microtubule stabilization in senescent intestinal epithelial cells

**DOI:** 10.1038/s41514-022-00097-8

**Published:** 2022-12-16

**Authors:** Siwei Chu, Ossama Moujaber, Serge Lemay, Ursula Stochaj

**Affiliations:** 1 Department of Physiology, McGill University, Montreal, Quebec H3G 1Y6 Canada; 2 Department of Medicine, Division of Nephrology, McGill University Health Centre, Montreal, QC Canada

**Keywords:** Senescence, Ageing

## Abstract

Intestinal epithelial cells are critical for gastrointestinal homeostasis. However, their function declines during aging. The aging-related loss of organ performance is largely driven by the increase in senescent cells. To date, the hallmarks and molecular mechanisms related to cellular senescence are not fully understood. Microtubules control epithelial functions, and we identified microtubule stabilization as a phenotypic marker of senescent intestinal epithelial cells. The senescence inducer determined the pathway to microtubule stabilization. Specifically, enhanced microtubule stability was associated with α-tubulin hyperacetylation or increased abundance of the microtubule-binding protein tau. We show further that overexpression of *MAPT*, which encodes tau, augmented microtubule stability in intestinal epithelial cells. Notably, pharmacological microtubule stabilization was sufficient to induce cellular senescence. Taken together, this study provides new insights into the molecular mechanisms that control epithelial cell homeostasis. Our results support the concept that microtubule stability serves as a critical cue to trigger intestinal epithelial cell senescence.

## Introduction

Gastrointestinal health is essential to the performance of other organs. This relationship is well documented for the gut-brain axis^1^. Epithelial cells separate the lumen of the intestine from the rest of the organism^2^. They limit the access of pathogens and other damaging materials to the underlying tissue^3^. The barrier function of the intestinal and other epithelia is controlled by the cytoskeleton. Especially the microtubule network plays an essential role in epithelial homeostasis^4,5^. For example, microtubules regulate cell shape and organization, polarization, and the proper interactions with neighboring cells^6,7^. Microtubules also provide tracks for intracellular transport, and participate in cell migration^8^, brush border formation and maintenance^9^, as well as apical epithelial cell extrusion^10^. Many of these functions rely on the dynamic properties of microtubules. Accordingly, changes in microtubule stability impact multiple aspects of intestinal cell physiology.

Aging is defined as the “progressive decline in physiological integrity”; it raises the susceptibility to death^11^. One key feature of aging is cellular senescence, which is characterized by the loss of proliferative potential and altered homeostasis^11,12^. Moreover, cellular senescence promotes aging, which is accompanied by marked changes at the cell, tissue, organ, and organismal levels^13^. The aging-related decline in epithelial barrier function is often accompanied by disease^14,15^. In humans, the impairment of intestinal barrier functions has been linked to neurodegeneration^16^. Thus, a better understanding of the biomarkers and pathways related to epithelial cell senescence is crucial to prevent or ameliorating the pathologies associated with the aging of epithelia.

Diverse pathways lead to cellular senescence, and various model systems uncovered cell type-specific differences in senescence biomarkers and phenotypes^17–21^. To date, the molecular mechanisms driving the senescence of mammalian epithelia are not fully defined. As such, it is unknown whether cytoskeletal reorganization and the underlying mechanisms are shared by senescent epithelial cells of different origins. It is also unclear whether different molecular pathways lead to a “senescent cytoskeleton”. Moreover, whether the organization of interphase microtubules impinges on cellular senescence remains largely unexplored. The current study was designed to address these knowledge gaps.

## Results

### Models to study senescence in intestinal epithelial cells

Sodium butyrate or lopinavir can induce senescence in renal proximal epithelial cells^20,22^. Here, we have adopted our methods to identify senescence-associated changes in intestinal epithelial cells (IECs). Both agents are physiologically relevant to intestinal epithelia. Butyrate produced by microbiota contributes to intestinal cell aging^23,24^, whereas the anti-retroviral drug lopinavir is used for HIV therapy. Lopinavir accumulates in intestinal cells and can disrupt the intestinal barrier function in vitro and in vivo^25,26^.

As different cell types vary in their sensitivity to senescence inducers^21^, our initial experiments determined the potential toxicity of butyrate and lopinavir for IECs (Supplementary Fig. 1). To this end, we measured the cellular viability for increasing concentrations of either agent. On the basis of the results shown in Supplementary Fig. 1, IECs were treated in subsequent experiments with 10 mM sodium butyrate for 5 days or with 30 µM lopinavir for 3 days. The selected concentrations diverge from the conditions we have developed for renal proximal epithelial cells^20^. These results underscore that the origin of cells determines the response to senescence triggers.

Additional experiments examined whether a subpopulation of the IECs underwent apoptosis upon the induction of senescence. To achieve this, floating and attached cells were evaluated for molecular markers of apoptosis. In particular, the loss of PARP1, and the cleavage of lamin A or caspase-3 were investigated. Supplementary Fig. 1 illustrates that both butyrate and lopinavir caused death for a subpopulation of cells. As apoptotic cells generally detach from the growing support, we conducted all following experiments with cells that remained adherent after treatment.

**Fig. 1:**
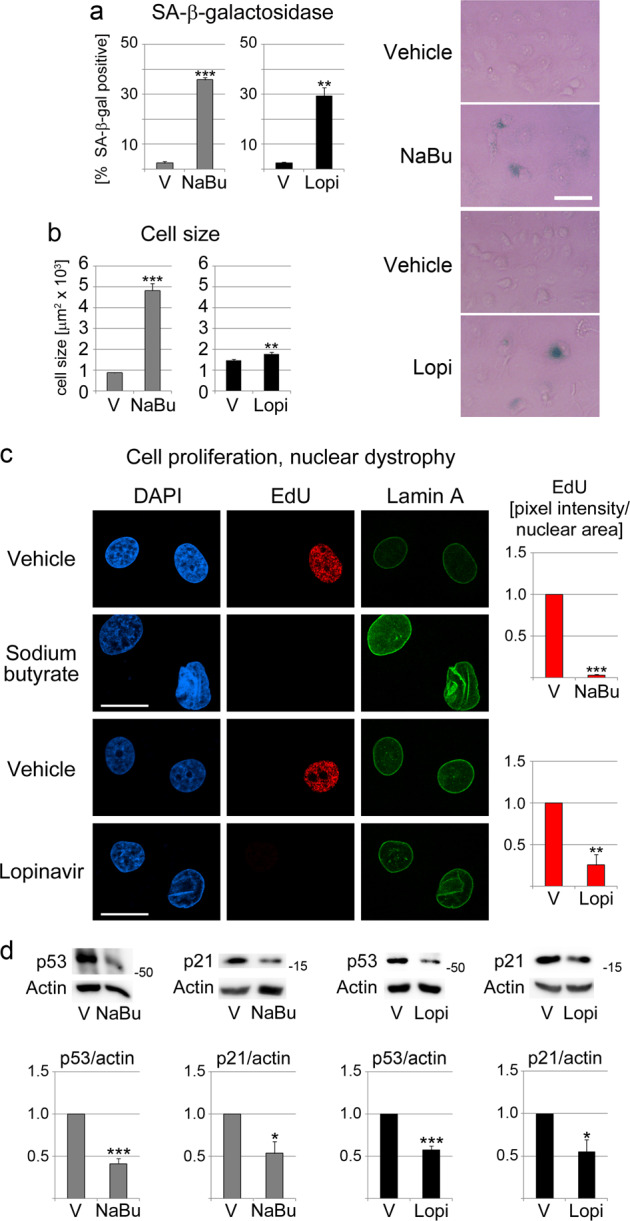
Sodium butyrate and lopinavir treatment induce senescence in intestinal epithelial cells (IECs). IECs were incubated for 5 days with 10 mM sodium butyrate (NaBu) or 3 days with 30 µM lopinavir (Lopi). Control cells were incubated with vehicle (V). Several hallmarks of cellular senescence were examined. **a** The number of cells positive for senescence-associated (SA) β-galactosidase activity was assessed in four (NaBu) or three (Lopi) independent experiments. For each data set, at least 100 cells were evaluated per condition. Representative images for SA-β-galactosidase activity are shown. The scale bar is 50 µm. **b** The cell size was measured for 129–241 cells/condition. **c** Cell proliferation (EdU incorporation) was scored in 128–181 cells/condition. Nuclear dystrophy was monitored by immunocytochemistry with antibodies against lamin A. Scale bar is 20 µm. **d** The abundance of p53 and p21 was reduced in senescent IECs. IECs attached to the growth surface were harvested, and the abundance of p53 and p21 was determined for crude extracts. Actin was used to normalize ECL signals to the vehicle controls. The molecular mass of marker proteins in kDa is shown at the right margin of the blots. Graphs represent results for three to six independent experiments. **a**–**d** Data are shown as average + SEM. Student’s *t*-test identified significant differences between vehicle controls and treated samples; **p < 0.01; ***p < 0.001.

The conditions we selected for IEC incubation triggered cellular senescence in accordance with acceptance criteria. Specifically, the treatments elevated senescence-associated β-galactosidase (SA-β-gal) activity, increased cell size, diminished cell proliferation, and induced dysmorphic nuclei (Fig. 1a–c). The initiation and establishment of cellular senescence is a multi-step process^21^. It can also be accompanied by changes in the abundance of p53 and p21. While the initiation of senescence is often associated with increased levels of p53 and p21, elevated p53 and p21 abundance is not required to maintain cellular senescence^21^. Indeed, both p53 and p21 abundance are diminished after the manifestation of growth arrest^21^. IECs treated with butyrate or lopinavir significantly reduced p53 and p21 levels (Fig. 1d). This corroborates the idea that cells have established a senescent phenotype.

Additional independent evidence for the induction of senescence by butyrate or lopinavir comes from the analysis of the cell secretome. To our knowledge, detailed studies on the senescence-related changes of secreted factors are not available for the IECs analyzed by us. This is relevant because the senescence-associated secretory phenotype (SASP) is determined by the senescence inducer and properties of the secreting cell. Nevertheless, several factors are commonly increased in the secretome of senescent mammalian cells^27^, including the proteins B23/nucleophosmin, GAPDH, and actin. We evaluated their levels in the growth medium of control and treated cells (Supplementary Fig. 2). Compared with vehicle controls, the extracellular abundance of the examined proteins increased upon incubation with butyrate or lopinavir. These results corroborate that butyrate and lopinavir serve as senescence inducers for IECs under our experimental conditions.

For several of the experiments discussed below, we challenged cells with sodium arsenite (here referred to as arsenite), which causes oxidative stress. The stressor was included because senescence compromises stress responses^22,28^. Relevant to the current study, several aspects of stress responses are regulated by the cytoskeleton^29,30^.

**Fig. 2:**
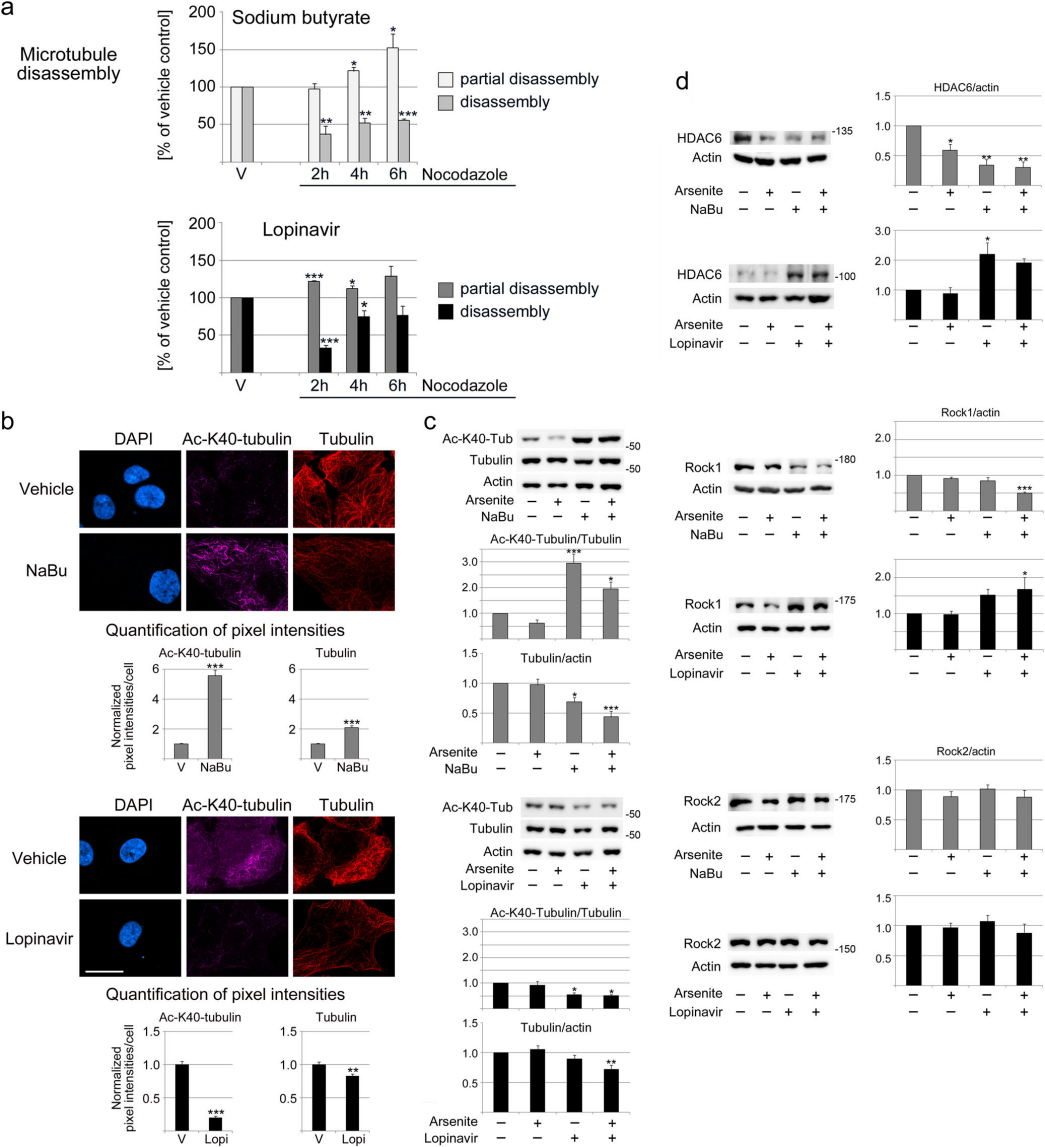
Senescent intestinal cells reorganize microtubules. **a** Microtubule stability was evaluated with nocodazole for IECs treated with vehicle (V), sodium butyrate (NaBu), or lopinavir. Disassembly or partial disassembly was scored^10^. Data were normalized to vehicle controls. Average + SEM are shown for three independent experiments. For each experiment, 100–157 cells were examined for every time point and condition. **b** Immunocytochemistry revealed changes in the abundance and organization of α-tubulin acetylated on K40 and total α-tubulin (Tubulin). DAPI stained nuclei; scale bar is 20 µm. Graphs depict pixel intensities/cells normalized to vehicle controls (V). At least 135 cells were assessed for each condition. Bars represent average + SEM. **c** Semiquantitative Western blotting determined the abundance of α-tubulin acetylated on K40 (Ac-K40-Tubulin) and total α-tubulin. Actin was used as a loading control. Between three and seven independent experiments were evaluated by Western blotting. **d** Western blotting determined the abundance of HDAC6, Rock1, and Rock2 for the conditions depicted. Graphs represent averages + SEM for 3–8 independent experiments. **a**, **b** Student’s *t*-test evaluated differences between control and senescent cells; *p < 0.05; **p < 0.01; ***p < 0.001. **c**, **d** The molecular masses of marker proteins in kDa are indicated at the right margin of blots. One-way ANOVA combined with Bonferroni correction identified significant differences between the vehicle control and treated cells. *p < 0.05; **p < 0.01; ***p < 0.001.

Our previous work on renal proximal epithelial cells uncovered cytoskeletal changes when these kidney cells undergo senescence^20^. Hence, we hypothesized that cytoskeletal reorganization is a shared feature of senescence in epithelia. In support of this hypothesis, senescent IECs displayed stabilized and rearranged microtubule filaments (Fig. 2a, b). Moreover, microtubules became more resistant to disassembly and less organized. At the same time, the overall cell morphology changed (Figs. 1 and 2b).

Posttranslational tubulin modifications regulate filament properties^31^, and lysine 40 (K40) acetylation of α-tubulin is associated with stable microtubules^32^. In IECs, K40 acetylation increased significantly with butyrate incubation (Fig. 2c). Notably, lopinavir treatment also stabilized microtubules, but K40 acetylation was diminished. This suggested alternative mechanisms for lopinavir-dependent microtubule stabilization in senescent IECs.

To identify the mechanisms that lead to microtubule stabilization in senescent IECs, we first examined HDAC6. HDAC6 is the major deacetylase for α-tubulin acetylated on K40. HDAC6 regulates microtubule acetylation and stability in the intestine and other organs^33^. Treatment with butyrate reduced HDAC6 abundance significantly (Fig. 2d). By contrast, lopinavir increased HDAC6 levels, which is consistent with the loss of K40 acetylation (Fig. 2c). The use of two unrelated antibodies verified that these differences were not due to limitations of the primary antibody (Supplementary Fig. 3).

Aside from HDAC6, Rock1 (Rho-associated protein kinase 1) and Rock2 also regulate cytoskeletal organization. Both kinases are implicated in aging and cellular senescence^20^. Importantly, Rock1 controls the intestinal barrier function^34^. In IECs, butyrate diminished Rock1 levels, whereas lopinavir had the opposite effect (Fig. 2d). These differences were heightened when senescent cells were stressed with arsenite. None of the conditions had marked effects on Rock2 (Fig. 2d).

To identify a possible link between Rock activity and K40 acetylation, IECs were incubated with different concentrations of the Rock inhibitor Y27632 for 3 days. The selected concentrations of Y27632 effectively inhibit Rock1 and Rock2 in mammalian cells^35,36^. Following Rock inhibition, crude cell extracts were assessed for K40 acetylation and α-tubulin levels. As shown in Supplementary Fig. 4, the changes in K40 acetylation and α-tubulin abundance were minor for all conditions tested. Accordingly, the kinase activities of Rock1 and Rock2 were not major modulators of K40 acetylation in IECs.

Collectively, the results in Fig. 2 provide quantitative evidence for senescence-associated changes in the microtubule cytoskeleton in IECs. Our data demonstrate that the senescence-related stabilization of microtubules is a shared feature of intestinal and renal epithelial cells^20^. Interestingly, the senescence inducer controls the pathway to microtubule stabilization.

### Microtubule stability increases in senescent fibroblasts

**Fig. 3:**
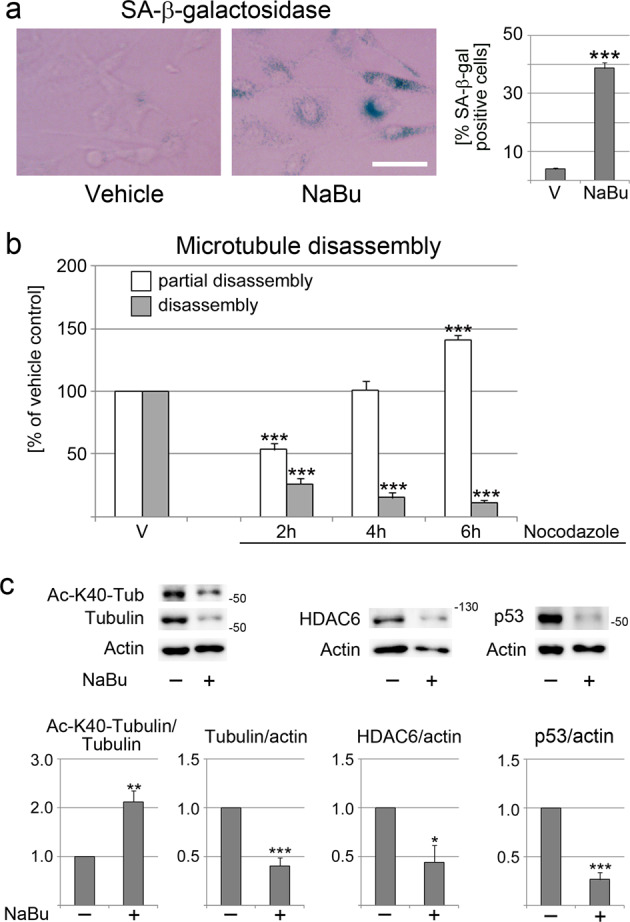
Microtubules become stabilized in senescent fibroblasts. **a** Incubation with 5 mM sodium butyrate for 5 days increased the number of SA-β-gal positive cells. Images depict SA-β-gal activities in cells incubated with vehicle or sodium butyrate (NaBu). The size bar is 50 µm. The graph shows the results of three independent experiments. A minimum of 100 cells were scored per condition for each experiment. Student’s *t*-test revealed significant differences between the vehicle (V) and sodium butyrate incubation (NaBu). ***p < 0.001. **b** Microtubule stability was evaluated with nocodazole, as described in Fig. 1. The assay uncovered a significant increase in microtubule stability in senescent fibroblasts. Student’s *t*-test was applied to uncover significant differences between control and butyrate-treated cells; ***p < 0.001. **c** Crude extracts prepared for control and senescent fibroblasts were assessed for K40-acetylated α-tubulin, α-tubulin, HDAC6, and p53 abundance. Numbers at the right margins of blots indicate the position of marker proteins in kD. Graphs represent averages + SEM for three to four independent experiments. Statistical evaluation was conducted with Student’s *t*-test. *p < 0.05; **p < 0.01; ***p < 0.001.

To determine whether cellular senescence is linked to microtubule stabilization in non-epithelial cells, we expanded our analyses to NIH3T3 fibroblasts. Butyrate caused senescence in approximately 40% of the cells under the conditions tested (Fig. 3a), while lopinavir led to massive cell death. Treatment of control and senescent fibroblasts with nocodazole uncovered a significant rise in microtubule stability when NIH3T3 cells became senescent (Fig. 3b). In senescent NIH3T3 fibroblasts, K40 α-tubulin acetylation increased, whereas the abundance of α-tubulin, HDAC6 and p53 diminished (Fig. 3c). Taken together, these results support the concept that a senescence-associated stabilization of microtubules is not limited to epithelial cells; it provides a senescence marker for multiple cell types.

### The microtubule-binding protein tau increases in abundance upon lopinavir-induced IEC senescence

The loss of K40 acetylation in lopinavir-treated IECs suggested alternative routes to microtubule stabilization. Candidates for these activities are microtubule-associated proteins (MAPs), a set of proteins that modulate microtubule stability. Tau, MAP1B, MAP2, and MAP4 are lattice-binding proteins that can stabilize microtubule filaments^37^. Tau also produces disease-associated aggregates and undergoes phase separation; this process is likely relevant to neurodegeneration^38^. To our knowledge, tau condensates outside of the CNS or in non-neuronal cells have not been reported so far.

Mammalian cells generate multiple tau isoforms; isoform production is cell type-specific and developmentally regulated^39^. Tau and its various isoforms have been extensively studied in neurons. Outside of the CNS, the health relevance of tau in the enteric nervous system is coming to light^40^. By contrast, the role of tau in non-neuronal cells remains poorly defined. Intracellular tau is present in the cytoplasm, and at least in part associated with microtubules. However, tau also resides in other cell compartments, where it may have microtubule-independent functions^41^.

**Fig. 4:**
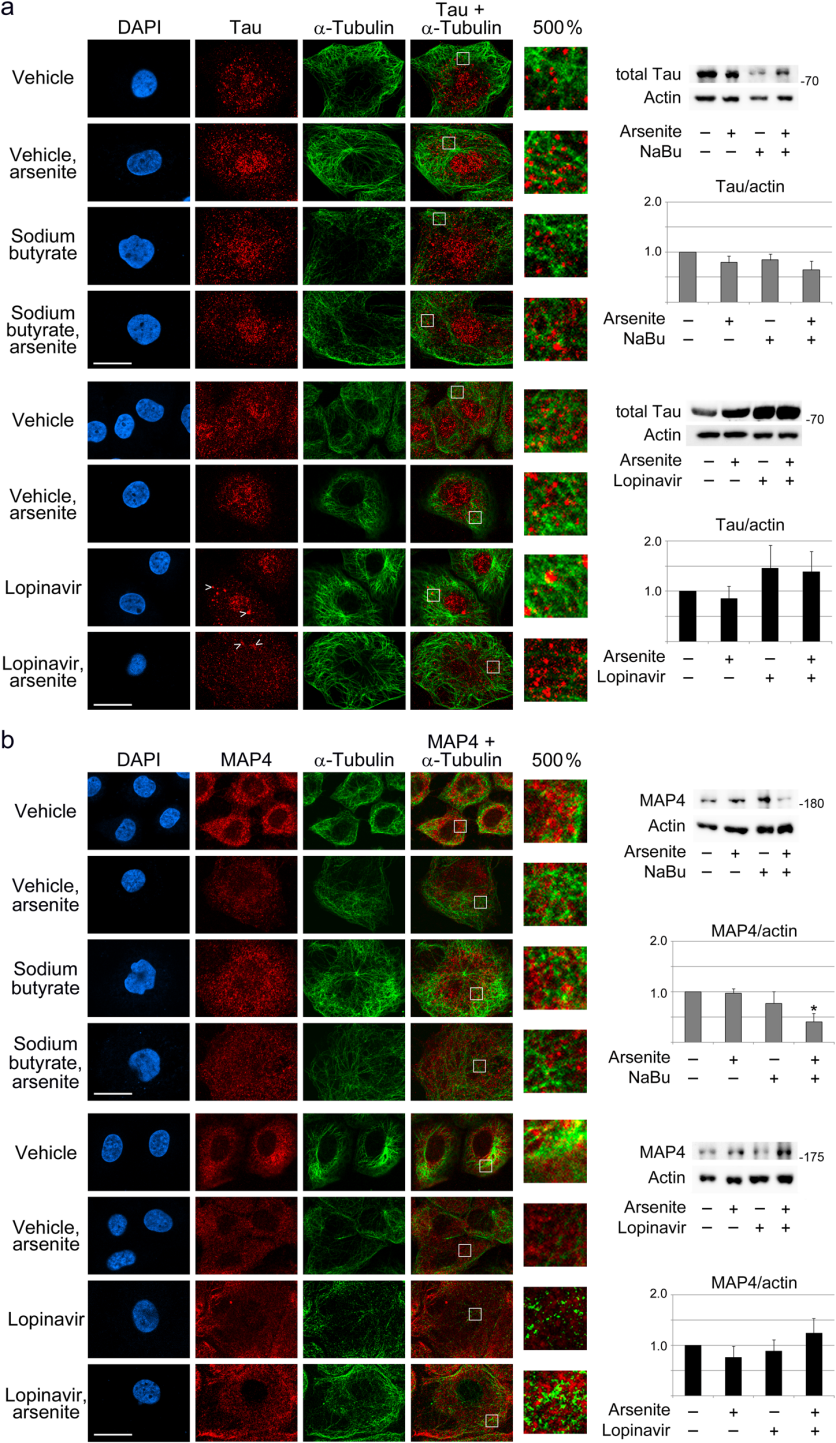
Cellular senescence and stress modulate the distribution and abundance of the microtubule-associated proteins tau and MAP4. IECs were incubated with arsenite, sodium butyrate (NaBu), or lopinavir as described in the Methods section. The subcellular distribution of tau (**a**) and MAP4 (**b**) were assessed by immunocytochemistry. The scale bar is 20 µm. The positions of several tau aggregates, located in the nucleus or cytoplasm, are marked (>). Selected regions, delimited by a white margin, were enlarged to 500%. To compare the distribution of α-tubulin, fluorescent signals were increased in a linear fashion uniformly over the entire panel in part a for sodium butyrate and in part b for lopinavir. Western blotting monitored the abundance of the 75 kDa form of tau (**a**) or of MAP4 (**b**); actin provided a loading reference. The molecular mass of marker proteins is indicated at the right margin of blots. Graphs represent average + SEM, with three to seven independent experiments for each data set. One-way ANOVA combined with Bonferroni correction was used for statistical evaluation; *p < 0.05.

Our study uncovered that tau abundance increased in IECs treated with lopinavir. While these changes were variable, the trend was clear for IECs incubated with lopinavir or lopinavir + arsenite (Fig. 4). As discussed below, tau levels significantly increased in the intestine of aging mice, supporting the idea that tau abundance is modulated during cellular senescence and organismal aging. Notably, the changes take place in cells and tissues outside of the nervous system.

We hypothesized that the subcellular localization of tau is dynamic and affected by alterations in cellular homeostasis. For example, stress or senescence may shift tau distribution towards the cell nucleus. Indeed, such a shift was observed for arsenite, butyrate, the combination butyrate + arsenite, and lopinavir (Fig. 4a). Quantitative image analyses substantiated the changes in subcellular tau distribution (Supplementary Fig. 5). The nucleocytoplasmic ratios significantly increased for tau when cells were incubated with butyrate or butyrate + arsenite. There was also a small increase in the nucleocytoplasmic ratio with lopinavir, but this trend was not significant. On the other hand, the combination of lopinavir and arsenite significantly reduced the nucleocytoplasmic ratio for tau.

The nuclear localization of tau could protect IECs against DNA damage^41^, which rises in several tissues during aging^42^. In line with this model, IECs increased the levels of γH2AX in nuclei, which indicated genomic DNA lesions. Specifically, IEC senescence elevated nuclear γH2AX to 136% (butyrate) or 142% (lopinavir) of their non-senescent counterparts. Interestingly, larger tau aggregates, suggestive of phase separation, were detected with lopinavir (Fig. 4a, marked with >), but less evident for butyrate.

Western blotting identified a 75 kDa tau species as a prominent isoform in IECs (Fig. 4a). Arsenite and butyrate slightly reduced the 75 kDa band, whereas tau levels increased with lopinavir. Since tau can stabilize microtubules, our results support the model that tau contributes to microtubule stabilization upon lopinavir-induced IEC senescence.

Collectively, our data substantiate the hypothesis that different senescence inducers trigger common and specific cellular responses. In lopinavir-treated IECs, senescence-associated microtubule stabilization is not linked to higher K40 acetylation of α-tubulin. However, microtubules can be stabilized –at least in part- through a rise in tau abundance.

MAP1B, MAP2, and MAP4 also regulate microtubule stability^37^. MAP4 is abundant in the gastrointestinal tract, especially in the small intestine^43^. As MAP4 protects microtubules from disassembly^44^, we compared the subcellular distribution and abundance of MAP4 in senescent and non-senescent IECs (Fig. 4b, Supplementary Fig. 5). MAP4 was mostly cytoplasmic for control conditions and lopinavir-treatment. Nuclear MAP4 slightly increased with arsenite, butyrate, and the combination of butyrate + arsenite. In parallel, total MAP4 concentrations, as determined by Western blotting, diminished when butyrate was present. The loss of MAP4 was significant for the combination treatment butyrate + arsenite (Fig. 4b). This may suggest that butyrate induced a portion of MAP4 to relocate to nuclei. Alternatively, MAP4 may have been preferentially degraded in the cytoplasm under these conditions. Lopinavir had no significant impact on the abundance of MAP4 (Fig. 4b). Interestingly, arsenite alone elevated MAP4 levels in the nucleus, whereas the opposite was observed for the combination of lopinavir + arsenite (Supplementary Fig. 5). Future experiments will have to explore the role of MAP4 in senescent IECs in more detail.

The evaluation of additional MAPs revealed that cellular senescence reduced MAP2 abundance; the effect was particularly pronounced for lopinavir (Supplementary Fig. 6). MAP1B is located in the nucleus and cytoplasm of HeLa cells^45^, but accumulated in the nuclei of IECs (Supplementary Fig. 7a). Western blotting detected multiple bands for MAP1B which were diminished by arsenite stress both in control and senescent cells (Supplementary Fig. 7b). However, cellular senescence alone did not change MAP1B abundance.

Taken together, our results show that microtubules in senescent IECs were not stabilized due to an increase in MAP1B, MAP2, or MAP4 abundance. This interpretation applies to senescence induced with butyrate or lopinavir.

### Lopinavir modulates the abundance of tau in epithelial cells of different origin

As discussed above, lopinavir caused IECs to undergo senescence. Our previous studies showed that lopinavir also triggers senescence in proximal tubule cells of the kidney^20^. Whether lopinavir affects the abundance of tau in these cells is not known. Supplementary Fig. 8 illustrates the effects of lopinavir on renal proximal epithelial cells. Western blotting detected an abundant tau species with an apparent molecular mass of 37 kDa (Tau37). This tau isoform increased in abundance with arsenite, lopinavir, and lopinavir + arsenite, but the trend did not reach statistical significance. Our results for IECs and renal proximal tubule cells show that lopinavir-induced senescence and oxidative stress modulate tau protein levels in epithelial cells that are derived from different origins.

### Pharmacological stabilization of microtubules induces cellular senescence

**Fig. 5:**
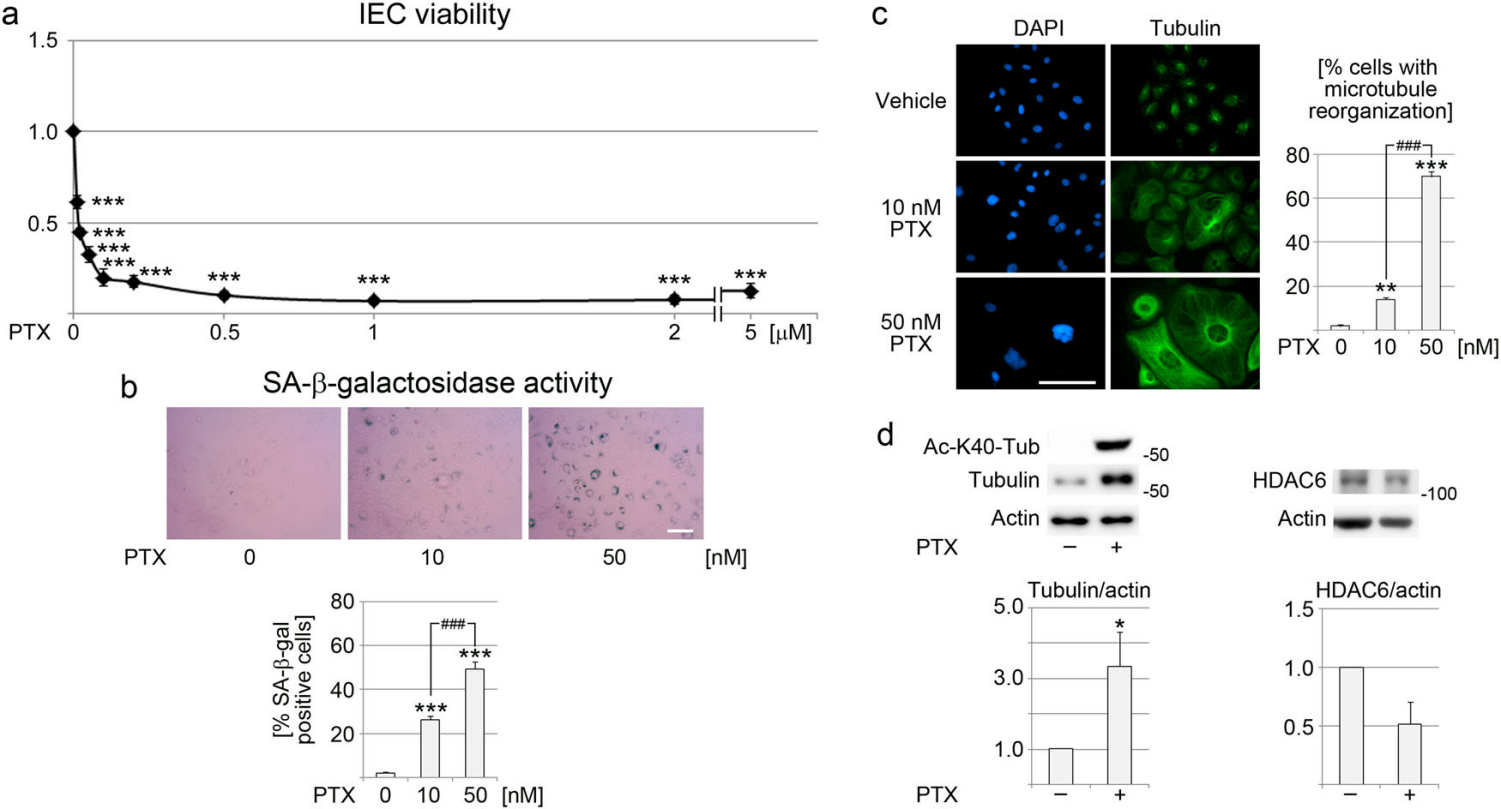
Paclitaxel triggers senescence in IECs. **a** IECs were treated with increasing concentrations of paclitaxel (PTX), as indicated. After a 5-day incubation period, cell viability was measured. The graph shows data from three independent experiments. One-way ANOVA combined with Bonferroni post-hoc correction revealed that all treatment groups differed significantly from the vehicle control. ***p < 0.001. **b** IECs were stained for SA-β-gal activity; the size bat is 100 µm. The number of SA-β-gal positive cells was determined for IECs incubated with vehicle, 10 nM, or 50 nM PTX for 5 days. The graph depicts the quantification of results for three independent experiments. At least 132 IECs were scored per condition for each experiment. One-way ANOVA with Bonferroni correction was performed for statistical evaluation. ***p < 0.001; ^###^*p* < 0.001. **c** IECs incubated for 5 days with vehicle, 10 nM, or 50 nM PTX were fixed and processed for immunostaining with antibodies against α-tubulin; the size bar is 100 µm. The percentage of cells with tubulin reorganization is shown in the graph. At least 100 cells were scored for each condition in every experiment. One-way ANOVA with Bonferroni correction identified significant differences. **p < 0.01; ***p < 0.001; ^###^*p* < 0.001. **d** After a 5-day treatment with 50 nM PTX, K40 acetylation, and the levels of α-tubulin and HDAC6 were measured in crude extracts. The molecular mass of marker proteins is shown in kDa at the right margin of the blots. Results were quantified for three to five independent experiments. Student’s *t*-test uncovered significant differences; *p < 0.05. Note that the profound increase in K40 acetylation was beyond the linear range of the imager, precluding quantification. Bars in all graphs show averages + SEM.

Paclitaxel (PTX) is a commonly used agent that stabilizes microtubules^46^. It was used here to determine whether microtubule stabilization can stimulate cells to undergo senescence. Initial experiments determined the optimal conditions for IEC treatment with PTX (Fig. 5a). Based on these results, further experiments were conducted with 10 nM and 50 nM PTX. Both conditions significantly increased the number of SA-β-gal positive cells; the rise was more pronounced for 50 nM as compared with 10 nM PTX (Fig. 5b). The PTX-induced senescence was associated with significant changes in microtubule organization (Fig. 5c). Furthermore, 50 nM PTX profoundly elevated the abundance of K40-acetylated and total α-tubulin. By contrast, incubation with PTX reduced HDAC6 abundance, but this trend did not reach statistical significance. Together, these data support the hypothesis that microtubule stabilization causes senescence in interphase IECs.

### *MAPT* overexpression stabilizes microtubules in IECs

**Fig. 6:**
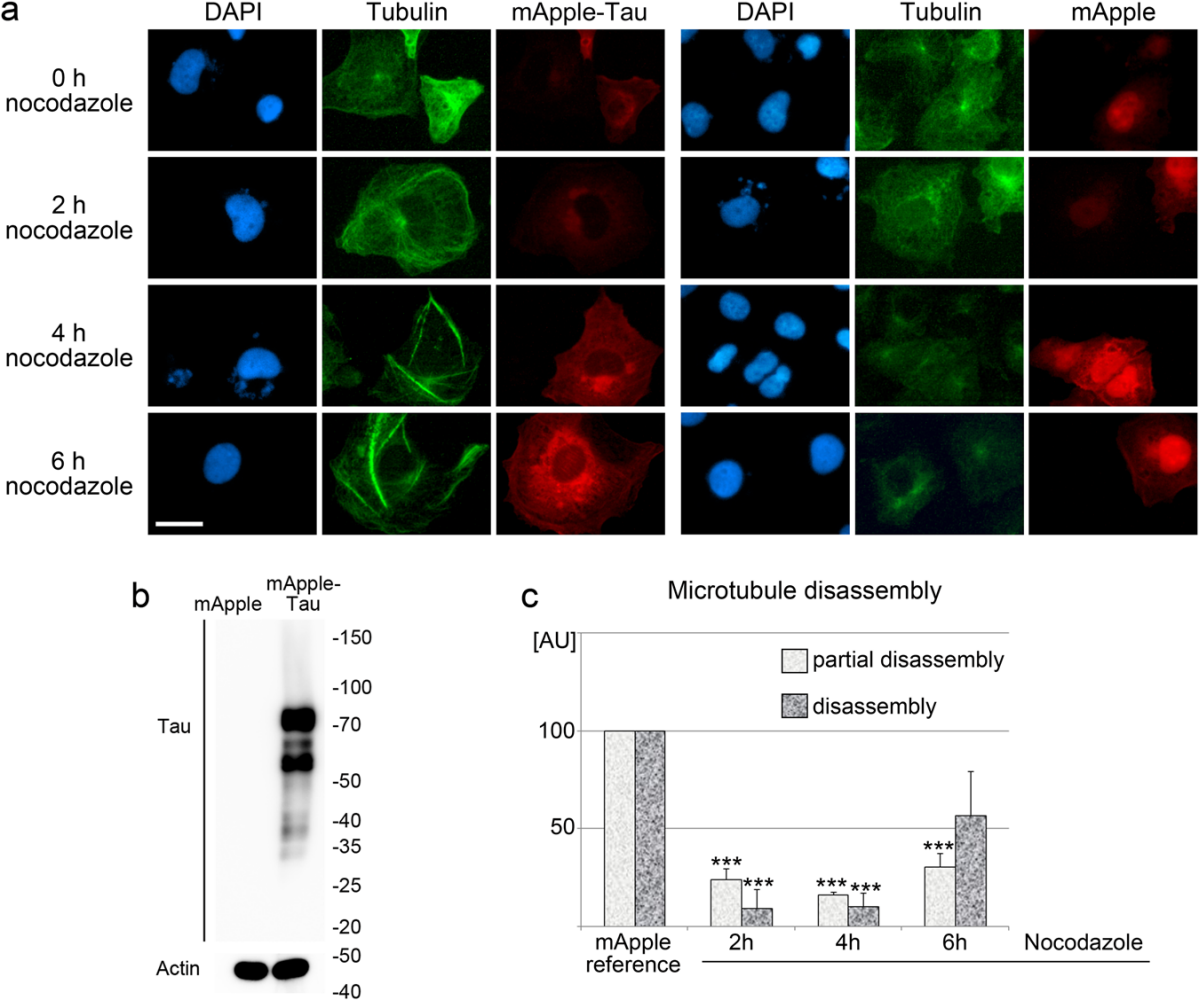
*MAPT* overexpression stabilizes microtubules in IECs. IECs were transiently transfected with plasmids encoding mApple-Tau or mApple alone. **a** IECs were fixed, and microtubules were visualized with antibodies against α-tubulin. Transfected cells were identified by red fluorescence emitted by the mApple-tag. Size bar is 20 µm. **b** Western blotting with antibodies against tau demonstrated the increased abundance upon transfection with the mApple-Tau construct. Actin was used as a loading control. The molecular mass of marker proteins in kDa is depicted at the right margin. **c** Microtubule stability was determined by incubation with nocodazole as described for Fig. 2. Microtubule disassembly was assessed for transfected cells. IECs producing the mApple-tag only provided the reference. Results were quantified for three independent experiments, each with at least 50 cells per condition. Each bar represents the average result + SEM. Student’s *t*-test uncovered significant differences between IECs producing mApple and mApple-Tau; **p < 0.01; ***p < 0.001.

The experiments discussed above are consistent with the idea that elevated tau levels contribute to microtubule stabilization in IECs. To obtain independent support for this concept, a mApple-Tau fusion was introduced into the cells (Fig. 6). IECs producing mApple-Tau or the mApple fluorescent tag were identified on the basis of their red fluorescence (Fig. 6a). Tau levels markedly increased upon the introduction of mApple-Tau when compared with mApple alone (Fig. 6b). Microtubule disassembly assays with nocodazole revealed that a rise in tau abundance stabilized microtubules (Fig. 6c). Accordingly, elevated tau levels stabilized microtubules in IECs.

### Tau abundance increases in the small intestine of aging mice

**Fig. 7:**
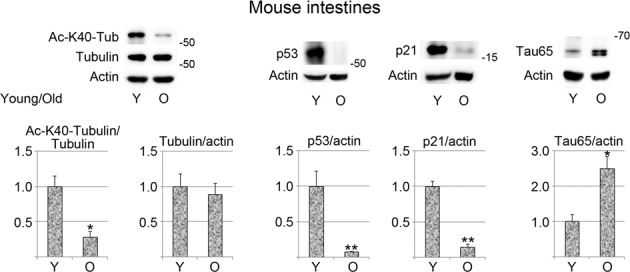
K40 α-tubulin acetylation, tau, and p53 abundance in mouse intestines are modulated by aging. Crude extracts were prepared for intestines from 2 months (*n* = 2, Young) or 14 months (*n* = 3, Old) mice. Western blotting evaluated the K40 acetylation of α-tubulin, total α-tubulin, HDAC6, p53, and tau levels. Actin served as a reference. Bars depict the averages of results + SEM. Student’s *t*-test was used for statistical evaluation. *p < 0.05; **p < 0.01.

As described above, IEC senescence induced with butyrate or lopinavir was associated with changes in K40 acetylation or, in the case of lopinavir, the elevation of tau levels. To obtain information on the in vivo relevance of these data, we extended our analyses to the intestine of experimental animals. To this end, the small intestine was harvested from young adults (Fig. 7; Young, Y) or mature mice (Old, O). Compared with young adult mice, the small intestines of older animals showed a significant reduction of K40 acetylation as well as p53 and p21 abundance. At the same time, tau was significantly elevated in the intestines of old animals. Interestingly, the molecular mass of tau detected for the small intestine had an apparent molecular mass of 65kD, whereas the tau isoforms upregulated in senescent IECs migrated at a position of 75 kD.

## Discussion

Cellular senescence drives organismal aging and disease. Complex and diverse routes lead to the initiation of cellular senescence. A senescent state can be acquired upon exposure to different inducers. Furthermore, the properties of a “senescent phenotype” are determined by the cell type or tissue undergoing senescence. Diverse markers are associated with senescence-related changes in cell physiology^21^, which is especially important for the proper identification of senescent cells.

Appropriate biomarkers and a detailed understanding of the pathways leading to senescence are prerequisites to ameliorating the senescence-associated derailment of cell functions. The current study expands knowledge in the field by focusing on microtubules in non-neuronal cells. To date, the role of the cytoskeleton for aging-related diseases has concentrated on neurons and their supporting cells in the central and peripheral nervous systems.

**Fig. 8:**
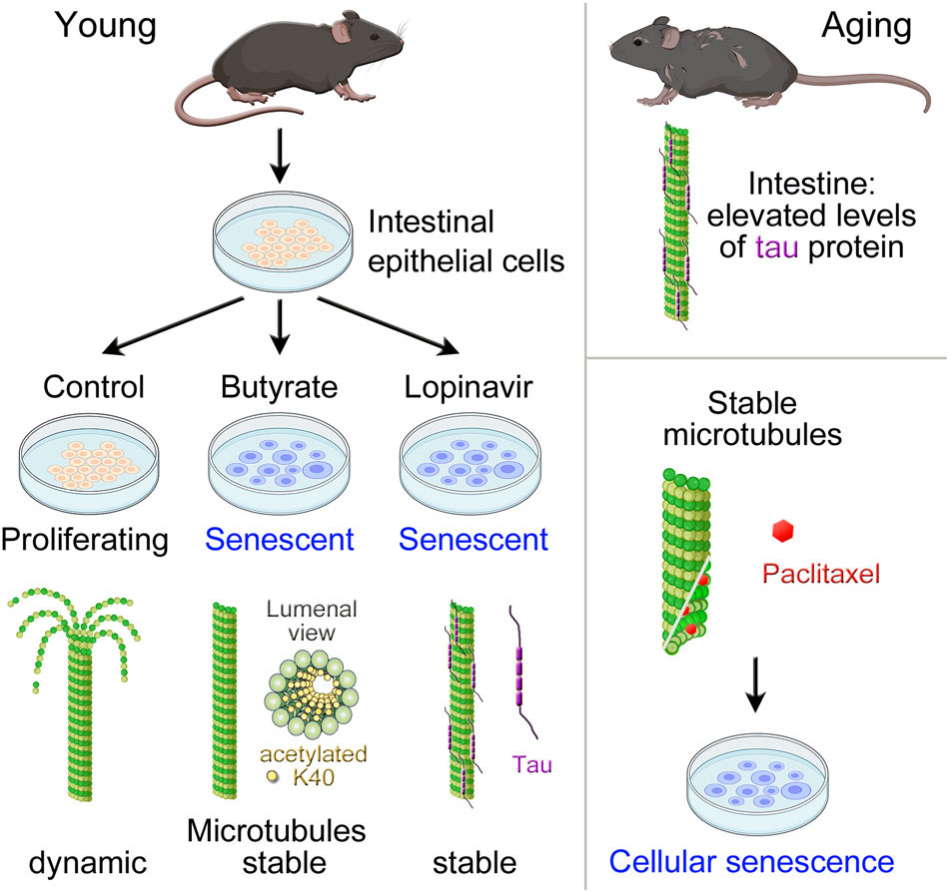
Model of senescence-associated changes in the microtubule cytoskeleton. Cellular senescence is accompanied by microtubule stabilization in IECs and other epithelial cells. The pathways to microtubule stabilization depend on the senescence inducer and the origin of epithelial cells. The mechanisms identified by us are associated with K40 α-tubulin acetylation or an increase in tau abundance. Microtubule stabilization and cellular senescence are interdependent processes, as microtubule stabilization triggers the induction of senescence.

Microtubules control the organization and function of epithelia^2,3^, but their role in cellular senescence has not been defined so far. We have now demonstrated that microtubule stabilization is a common denominator of IEC senescence, independent of the senescence inducer (summarized in Table 1 and Fig. 8). This property is shared by epithelial cells that originate from different organs, such as the intestine and kidney. Accordingly, the current study identified microtubule stabilization as a “hallmark of aging” for epithelial cells. As we have shown, microtubule stabilization is also a feature of senescent fibroblasts. Future experiments will examine whether the senescence-induced microtubule stabilization extends to additional cell types.

**Table 1:** Summary of the senescence-induced changes relevant to the microtubule cytoskeleton.

Parameter	Sodium butyrate	Lopinavir
Microtubule stability	increased	increased
K40 α-tubulin acetylation	increased	reduced
HDAC6 abundance	reduced	increased
Rock1 abundance	reduced	increased
Rock2 abundance	no change	no change
Tau localization	N + C, increased in N	slightly increased in N, aggregates in N and C
Tau abundance	slightly reduced	increased
Nuclear γH2AX	increased	increased
MAP4 localization	N + C, increased in N	no change
MAP4 abundance	slightly reduced	no change
MAP2 abundance	slightly reduced	reduced
MAP1B abundance	no change	no change

*N* nucleus, *C* cytoplasm, *N* *+* *C* nucleus and cytoplasm.

Although a general attribute of senescent epithelial cells, our work revealed that there is no uniform route to microtubule stabilization during senescence. This is consistent with the diverse effects of senescence inducers on cell physiology. Our study uncovered two distinct mechanisms that increase microtubule stability under conditions of cellular senescence. The first route involves changes in the post-translational modification of tubulin. In particular, K40 acetylation, a marker for stable microtubules, is modulated by cellular senescence. We have linked the senescence-induced increase in K40 acetylation to changes in the levels of HDAC6. The pathway operates when IECs are treated with butyrate. The second scenario applies to lopinavir-dependent senescence. The route is accompanied by elevated levels of the microtubule-binding protein tau. Overexpression of the *MAPT* gene verified that increasing the abundance of tau indeed led to more stable microtubules. This is relevant to the mechanisms driving cellular senescence, as tau has also microtubule-independent functions.

Notably, determined by the cell line or organism analyzed, tau species varied in their electrophoretic mobility. Our observation is consistent with the production of tau isoforms that is dependent on the species, cell type, as well as the developmental and physiological states of the cell^39,47,48^. Furthermore, numerous posttranslational modifications have been identified for tau, some of which can lead to marked changes in its molecular mass. Future studies will have to define how individual tau isoforms and their posttranslational modifications contribute to cellular senescence and aging outside of the nervous system.

Although outside of the focus of our study, we can speculate on the divergence of pathways that stabilize microtubules upon treatment with butyrate or lopinavir. For example, unlike HDAC6, butyrate inhibits a variety of other HDACs^49^. This has potentially marked effects on the expression of target genes that control the level of K40 acetylation. On the other hand, lopinavir inhibits the protease ZMPSTE24, which is involved in the processing of lamin A^50^. The excessive production of pre-lamin A, generated upon inhibition of ZMPSTE24, may trigger a stress response that ultimately induces cellular senescence^51^. Lamin A participates in numerous physical interactions^52^. For example, lamins serve as hubs that regulate diverse signaling events, which may control the production of tau. These interactions could regulate tau abundance through transcription of the *MAPT* gene or changes in the stability of tau transcripts^47^.

Outside of the nervous system, the role of tau in microtubule organization is beginning to emerge. One recent example includes kidney podocytes^53^. Our work further emphasizes the importance of tau for microtubule organization and homeostasis in non-neuronal cells. This study goes beyond the role of tau for cellular homeostasis, as we identified previously unexplored contributions of MAPs to cellular senescence. Long-term, this could open new opportunities for the detection of senescent cells.

Interestingly, we have shown a reciprocal relationship between microtubule stabilization and cellular senescence. On one hand, cellular senescence is accompanied by microtubule stabilization. On the other hand, microtubule stabilization is a driver of IEC senescence (Fig. 8). Thus, we identified a previously unknown mechanism that promotes epithelial cell senescence through microtubule stabilization. This information is especially relevant in the context of therapeutic treatments that employ modulators of microtubule stability, such as therapies for neurodegeneration or cancer^54,55^.

## Methods

### Growth and treatment of IEC-18, NIH3T3, and LLC-PK1 cells

IEC-18 cells^56^ (here referred to as IECs) were kindly provided from Dr. J Rak (McGill University, Montreal). The cells were grown in Dulbecco’s modified eagle medium, supplemented with 5% bovine calf serum, 10 µg/ml insulin (Sigma), and 1% penicillin/streptomycin. To induce cellular senescence, cells were treated with 10 mM sodium butyrate (Sigma) for 5 days; controls were incubated with the vehicle water. Alternatively, cellular senescence was induced by treatment with 30 μM lopinavir (Selleck) for 3 days; control cells received the vehicle DMSO. Oxidative stress was generated by a 30-min treatment with 0.5 mM sodium arsenite in growth medium. NIH3T3 fibroblasts and LLC-PK1 renal proximal tubule cells were grown under standard conditions^20^. Senescence induction with lopinavir and arsenite treatment of LLC-PK1 cells followed our published protocol^20^. Cultured cells were regularly tested for mycoplasma contamination with a commercially available kit (Applied Biological Materials, abm, Richmond, BC). All cell lines used in this study scored negative for mycoplasma.

### Cell viability assay

Cell viability was monitored with a resazurin (Acros Organics) reduction assay^57^.

### Evaluation of the secretome

To induce senescence, IECs were grown in 24-well plates and incubated with sodium butyrate or lopinavir, as described above. Controls were treated with the appropriate vehicle only. The growth medium was collected at day 5 (vehicle, sodium butyrate) or day 3 (vehicle, lopinavir), centrifuged at room temperature for 5 min (15,871 × *g*, microfuge) and stored at −70 °C for further analysis. For Western blotting, supernatants were precipitated with 10% TCA (final concentration) for 20 min on ice. Samples were further processed as published^20^. The number of cells in each well was determined by staining with crystal violet. In brief, after removal of the growth medium, cells were rinsed with PBS and fixed for 15 min with cold methanol (−20 °C). Following fixation, cells were stained with 0.5% crystal violet (Fisher Scientific) in 20% methanol for 10 min at room temperature. Samples were washed with PBS and dried overnight at room temperature, while protected from light. Cells were located with a UNICO IV900 microscope (10× objective) and photographed with an MU300 AmScope camera. For each well, images were acquired for 10 randomly selected fields. The number of cells per image was automatically determined with ImageJ. The identification of cells was verified by manual inspection and corrected if necessary. Based on the cell counts for the fields, the number of cells was calculated for individual wells. To analyze SASP components, sample loading was normalized to the number of cells present in each well (Supplementary Fig. 2).

### Treatment with paclitaxel

Paclitaxel (LC laboratories) was dissolved in DMSO; the vehicle was present in all samples at a final concentration of 0.3%. IECs were treated with vehicle or 10 nM to 5 μM paclitaxel for five days. Following treatment, cell viability was measured with the resazurin assay. To evaluate the effects on cellular senescence, IECs were treated with vehicle, 10 nM, or 50 nM paclitaxel for five days, followed by SA-β-Gal staining. For Western blotting, cells were incubated with vehicle or 50 nM paclitaxel for five days.

### Pharmacological inhibition of Rock activity

IECs were incubated with different concentrations of the Rock inhibitor Y27632 (Axon Medchem) for 3 days. Y27632 inhibits Rock1 and Rock2 in IECs^58^. The selected final concentrations of Y27632 efficiently inhibit Rock kinase activities in mammalian cells^35,36^.

### Evaluation of senescence-associated β-galactosidase and cell proliferation

The protocols to monitor SA-β-gal activity and cell proliferation with 5-ethynyl-2’-deoxyuridine (EdU) have been published^20^. At least 100 cells/conditions were assessed for each independent experiment.

### Microscopy

Images were acquired with a Zeiss LSM780 and a 63× objective as published^20^ or with a Nikon Optiphot and a 40× objective. Cells producing SA-β-gal were imaged with a Leica Galen III microscope and a 20× objective. For individual experiments, settings were identical for all conditions.

### Quantification of cell size, α-tubulin, and K40-acetyl-α-tubulin

For the quantification of fluorescence signals, images were acquired with a Zeiss LSM780 and a 20× objective. The pinhole was set to generate 15 µm thick sections. Identical settings were used for imaging of control and treated cells. Measurements were performed with MetaXpress software (Molecular Devices, San Jose, CA, USA, version 5.0.0.20), applying the cell scoring module and the regions measurements function. Each image was corrected for background fluorescence, followed by quantification of pixel intensities. At least 100 cells were analyzed per condition for every experiment.

### Quantification of nucleocytoplasmic protein distribution

To measure fluorescence intensities in the nucleus and cytoplasm, images were acquired with a Zeiss LSM780 and a 63× objective. A pixel size of 0.13 µm × 0.13 µm was used for all conditions. Images were analyzed with MetaXpress software and the Multi-wavelength Translocation module, as published by us^59^. In brief, following background correction, the size of nuclear and cytoplasmic compartments and their fluorescence intensities were measured. Correct segmentation was verified by visual inspection during image analysis. Cells improperly segmented were excluded from further analysis.

### Microtubule depolymerization

For microtubule depolymerization, IECs were treated with 500 nM nocodazole for 2, 4, and 6 h. Microtubule depolymerization was scored as described^20^.

### Immunocytochemistry

We have published detailed protocols for image acquisition and image quantification^20,22^.

### Plasmids and transfection

The plasmid encodingm mApple-Tau was obtained from Addgene (#54925).

The control construct coding for the mApple tag was generated from plasmid #54925 by dropping out the tau-coding region and subsequent autoligation. Transient transfections were performed with the K2 transfection kit (Biontex) according to the manufacturer’s protocol. Microtubule stability was analyzed 24 h after transfection.

### Western blotting

The preparation of crude extracts and Western blotting was performed as described^20^. Table 2 depicts the primary antibodies used for Western blotting. An initial set of experiments determined whether actin provided an appropriate loading control for the comparison of non-senescent and senescent IECs. Staining with Coomassie GelCode® Blue (Pierce) according to the supplier’s protocol assessed the protein blotted to PVDF filters. Colorimetric signals were measured (ChemiDoc™, BioRad), and the same filter was processed for Western blotting with actin-specific antibodies. For comparison, blots were also evaluated with antibodies against GAPDH, which is frequently used to monitor gel loading. Results in Supplementary Fig. 9 showed that actin, but not GAPDH, provided a proper loading control for IECs. Based on these results, actin was used as reference throughout the study.

Actin migrated with an apparent molecular mass of 46 kDa for all Western blots depicted in this study. Blots from the same experiment were used for quantification for each experimental data set. Western blot images were processed in Photoshop CS4 Extended, version 11.0. All blots were derived from the same experiment and processed in parallel. Unprocessed scans of the most important blots are included in Supplementary Fig. 10–13.

### Experimental animals, mouse intestines

Work with experimental mice has been approved by the McGill University Health Centre Animal Care Committee (Animal Use Protocol #MUHC-7602). Intestines were harvested from BL/6 mice that were 2 months (referred to as young) or 14 months of age (referred to as old). Intestines were snap frozen and kept at −70 °C. Samples were homogenized with a Bio-Gen PRO200 Homogenizer (Pro Scientific) in 0.5× concentrated gel sample buffer supplemented with protease and phosphatase inhibitors^10^. Samples were incubated for 15 min at 95 °C and sonicated twice for 5 min at room temperature (Dare flow, 80 W). Debris was removed by centrifugation at room temperature (3 min, 9391×*g*, microfuge). Supernatants were TCA-precipitated and analyzed by Western blotting.

### Primary and secondary antibodies

The source and dilution of primary antibodies are listed in Table 2.

**Table 2:** Sources and dilution of primary antibodies for Western blotting and immunocytochemistry.

Antigen	Source of antibody	Dilution for Western blotting	Dilution for immunocytochemistry
p53	Cell Signaling Technology; #2524	1:1000	NA
p21	My BioSource, MBS8539971	1:500	NA
B23/nucleophosmin	Cell Signaling Technology; #3542	1:2000	NA
K40 acetylated α-tubulin	Sigma, #T7451	1:10,000	1:2000
Total α-tubulin (mouse)	Santa Cruz Biotechnology, sc-5286	1:2000	1:500
Total α-tubulin (rabbit)	Abcam, ab15246	NA	1:400
HDAC6 (mouse)	Santa Cruz, sc-28386	1:500	NA
HDAC6 (rabbit)	Novusbio, NBP1-45611	1:250	NA
Rock1	Santa Cruz Biotechnology, sc-6056	1:500	NA
Rock2	Santa Cruz Biotechnology, sc-398519	1:500	NA
Tau	ThermoFisher, HT7 (MN1000)	1:1000	NA
Tau	Santa Cruz Biotechnology, sc-3904176	1:1000	NA
Tau	ABclonal, A1103	1:2000	NA
Tau	Sigma, SP70 (SAB5500182)	NA	1:200
MAP1B	Santa Cruz Biotechnology, sc-365668	1:1000	1:500
MAP2	Sigma, M4403	1:1000	1:500
MAP4	Santa Cruz Biotechnology, sc-390286	1:1000	1:100
Actin	Chemicon, MAB1501	1:100,000	NA
GAPDH	Santa Cruz Biotechnology, sc-32233	1:2000	NA
Lamin A	Santa Cruz Biotechnology, sc-20680	NA	1:500
Cleaved lamin A	Cell Signaling Technology; #2031	1:1000	NA
Cleaved caspase 3	Cell Signaling Technology; #9661	1:1000	NA
Lamin B	Santa Cruz Biotechnology, sc-6216	NA	1:1000

Affinity purified Alexa647, Cy3-, or Alexa488- labeled secondary antibodies were purchased from Jackson ImmunoResearch. Alexa Fluor® 647-conjugated (anti-mouse, 715-605-150; anti-goat, 705-605-147); Cy3-conjugated (anti-mouse, 715-165-150; anti-rabbit, 711-165-152), Alexa Fluor® 488-conjugated (anti-rabbit, 711-545-152). They were used at the following dilutions: Alexa Fluor® 647-conjugated (excitation/emission peaks: 651 nm/667 nm), 1:400; Alexa Fluor® 488-conjugated (excitation/emission peaks: 493 nm/519 nm), 1:400; Cy3 (excitation/emission peaks: 554 nm/568 nm), 1:500. Affinity-purified HRP-conjugated secondary antibodies were obtained from Jackson ImmunoResearch; anti-mouse (715-035-150; 115-035-071; 115-035-174); anti-rabbit (711-035-152, 111-036-046, 211-032-171), anti-goat (705-035-147). They were diluted at 1:2000. To minimize signals obtained for tissue-derived antibodies, samples prepared for mouse intestines were probed with secondary antibodies specific for IgG light chains or Fc fragments.

### Statistics

Statistical evaluation was performed with a two-tailed Student’s t-test for two groups. For more than two groups, One-way ANOVA combined with Bonferroni correction was conducted. Results are shown for pairwise comparisons between the vehicle control and the treated sample. Significant differences are marked with * for *p* < 0.05, ** for *p* < 0.01, *** for *p* < 0.001, or ### for *p* < 0.001.

## Supplementary information

Supplementary information

## Data Availability

The datasets generated and/or analyzed during this study are available from the corresponding author on reasonable request.
