# Supplementary material for: Multiple pathways promote microtubule stabilization in senescent intestinal epithelial cells
Source: NPJ Aging. 2022 Dec 16;8(1):16. doi: 10.1038/s41514-022-00097-8 (PMC9758230; doi:10.1038/s41514-022-00097-8)
Supplement: Supplementary file 1 — Supplementary information [file 41514_2022_97_MOESM1_ESM.pdf]

Supplementary information

Multiple pathways promote microtubule stabilization in senescent intestinal epithelial cells

Siwei Chu, Ossama Moujaber, Serge Lemay, and Ursula Stochaj
McGill University, Montreal, Canada

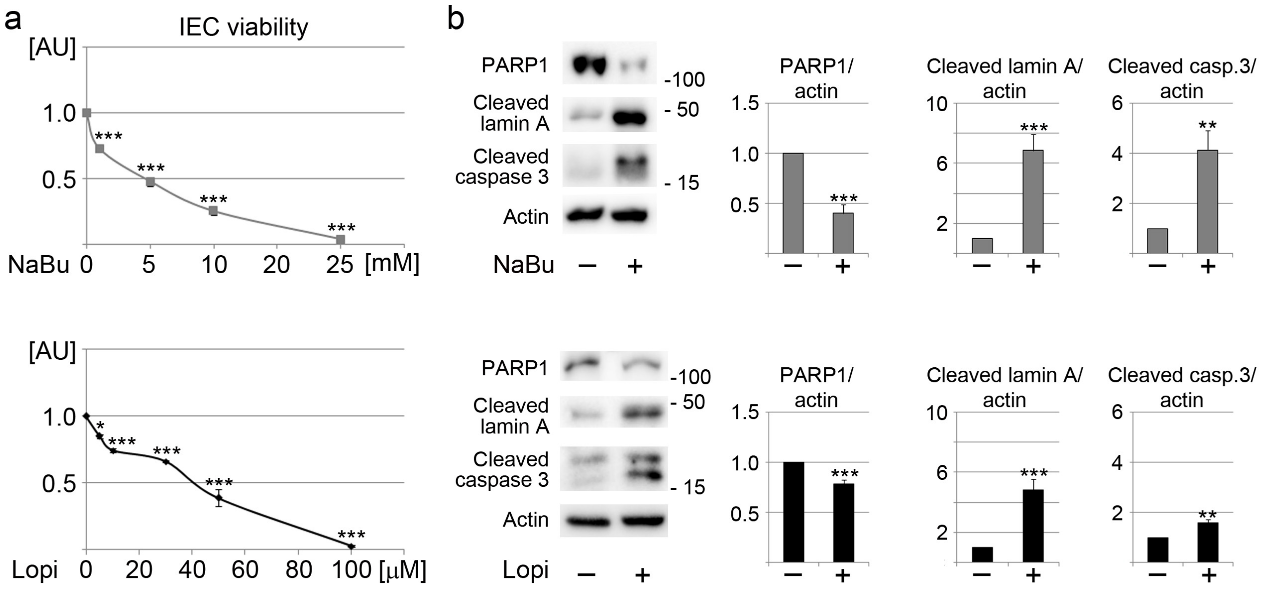

Supplementary Figure 1. Effects of sodium butyrate and lopinavir on the viability of intestinal epithelial cells (IECs). (a) IECs were incubated with increasing concentrations of sodium butyrate (NaBu, 5 days) or lopinavir (Lopi, 3 days) as depicted in the graphs. Cell viability was determined with a resazurin-based assay. Results are shown for three to four independent experiments. Significant differences were identified with One-way ANOVA combined with Bonferroni correction. *, $p < 0.05$; ***, $p < 0.001$. (b) Following the incubation with sodium butyrate (10 mM, 5 days) or lopinavir (30 µM, 3 days) floating and attached cells were combined for further analysis. Crude extracts were evaluated for markers of apoptosis. Representative examples of Western blots are shown; numbers on the right side represent the molecular mass of marker proteins in kDa. Graphs depict the average of results +SEM results for at least 5 independent experiments. Statistical evaluation was performed with Student's t-test; **, $p < 0.01$; ***, $p < 0.001$.

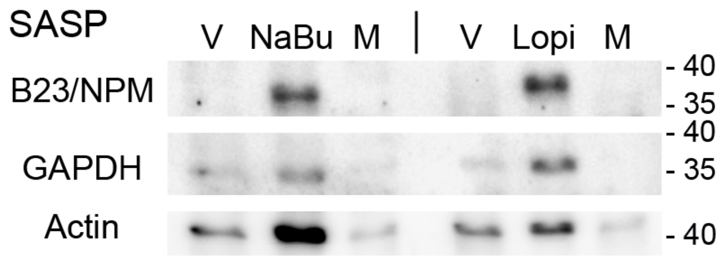

Supplementary Figure 2. Impact of sodium butyrate and lopinavir on the IEC secretome. IECs were incubated with vehicle (V), sodium butyrate (NaBu) or lopinavir (Lopi). Following treatment, the growth media were collected and examined for the presence of B23/nucleophosmin (B23/NPM), GAPDH, and actin. The loading was normalized to the number of cells that produced the secretome. For comparison, the growth medium alone (M) was analyzed in parallel. The position of marker proteins (in kDa) is depicted at the right margin. The Western blots are representative results of four independent experiments. The elevated abundance of B23/nucleophosmin (B23/NPM), GAPDH, and actin in the secretome is consistent with a senescence-associated secretory phenotype (SASP).

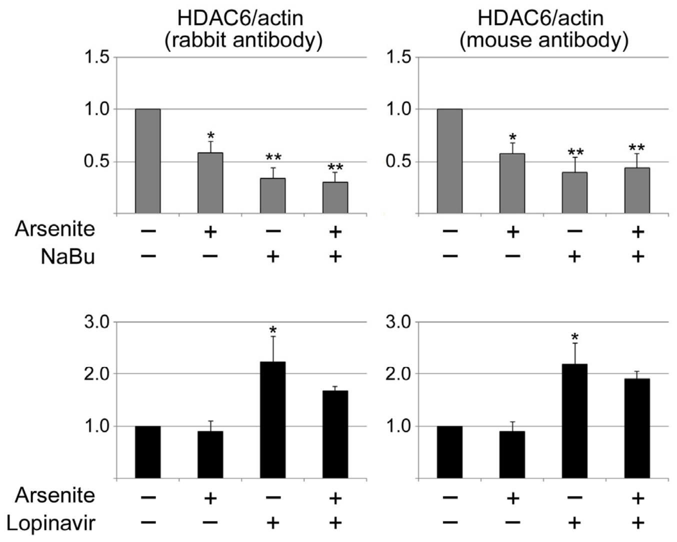

Supplementary Figure 3. Senescence-associated changes in HDAC6 abundance. Western blot results for HDAC6 were consistent for two unrelated antibodies, produced in different hosts (rabbit, mouse). Actin served as loading control. Results were normalized to the vehicle control. Graphs show average +SEM for at least three independent experiments. Statistical evaluation was performed with One-way ANOVA and Bonferroni correction. *, $p<0.05$; **, $p<0.01$. Both antibodies revealed that HDAC6 levels were reduced by sodium butyrate treatment, but increased with lopinavir.

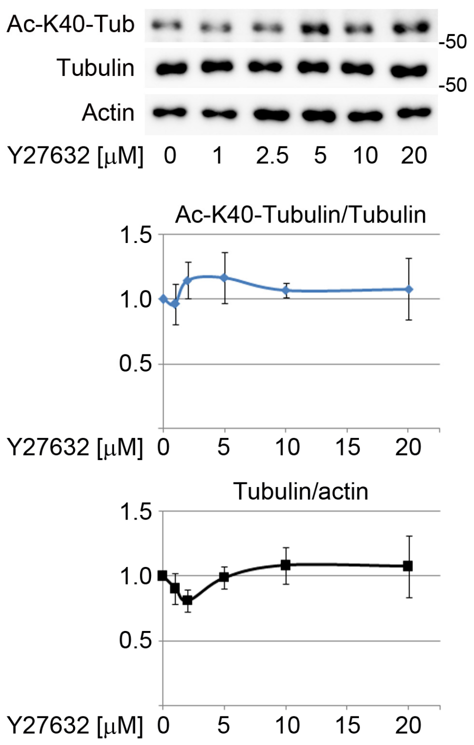

Supplementary Figure 4. Effects of the Rock inhibitor Y27632 on K40 α-tubulin acetylation in IECs. IECs were treated for 3 days with the Rock inhibitor Y27632 at the final concentrations indicated. Western blotting of crude extracts evaluated the acetylation of K40 and the abundance of α-tubulin. The molecular mass (in kDa) of marker proteins is depicted at the right margin. Actin was used as the loading reference. Graphs represent the quantification of 3 to 5 independent experiments. Results were normalized to the vehicle control; data are depicted as average ±SEM. One-way ANOVA combined with Bonferroni post-hoc test did not show significant differences for pairwise comparisons.

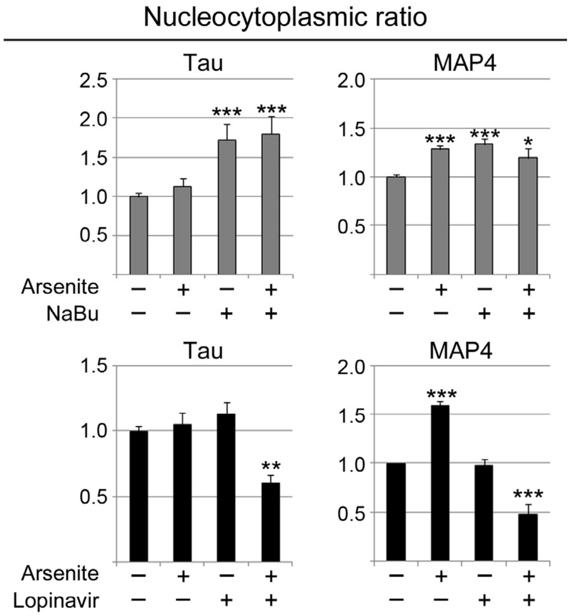

Supplementary Figure 5. Nucleocytoplasmic distribution of tau and MAP4 in control and senescent IECs. IECs were incubated with sodium butyrate (NaBu) or lopinavir and exposed to arsenite as indicated. Following treatment samples were fixed and images were acquired by confocal microscopy. The nucleocytoplasmic ratio of pixel intensities was quantified with MetaXpress software (see Methods for details). At least 8 cells were assessed for each condition; each bar represents the data average +SEM. Statistical evaluation was performed with One-way ANOVA combined with Bonferroni correction, with pairwise comparisons to the untreated control. *, $p<0.05$; **, $p<0.01$; ***, $p<0.001$.

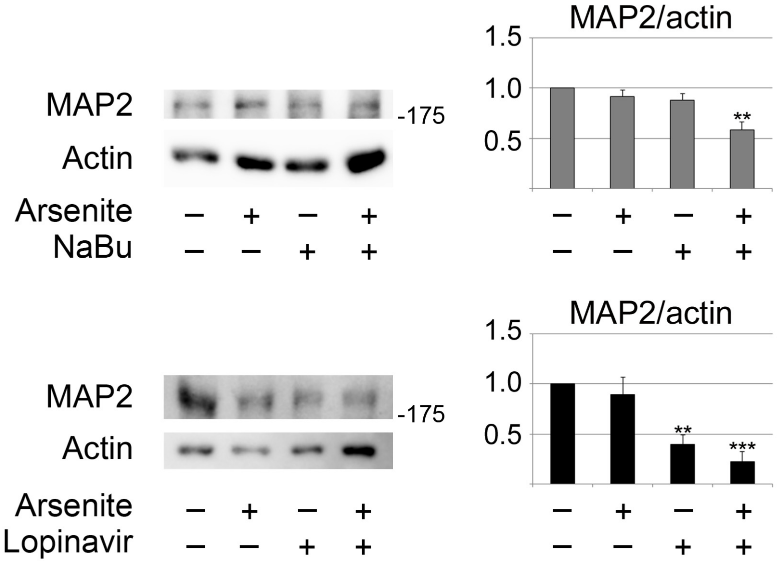

Supplementary Figure 6. Cellular senescence reduces the abundance of MAP2. IECs were incubated with arsenite, sodium butyrate (NaBu), or lopinavir as indicated. Crude extracts were assessed for the abundance of MAP2; actin provided the loading control. The molecular mass of marker proteins in kDa is shown at the right margin of the blots. Graphs depict the average +SEM for two to three independent experiments. One-way ANOVA combined with Bonferroni correction identified significant differences; **, $p<0.01$; ***, $p<0.001$.

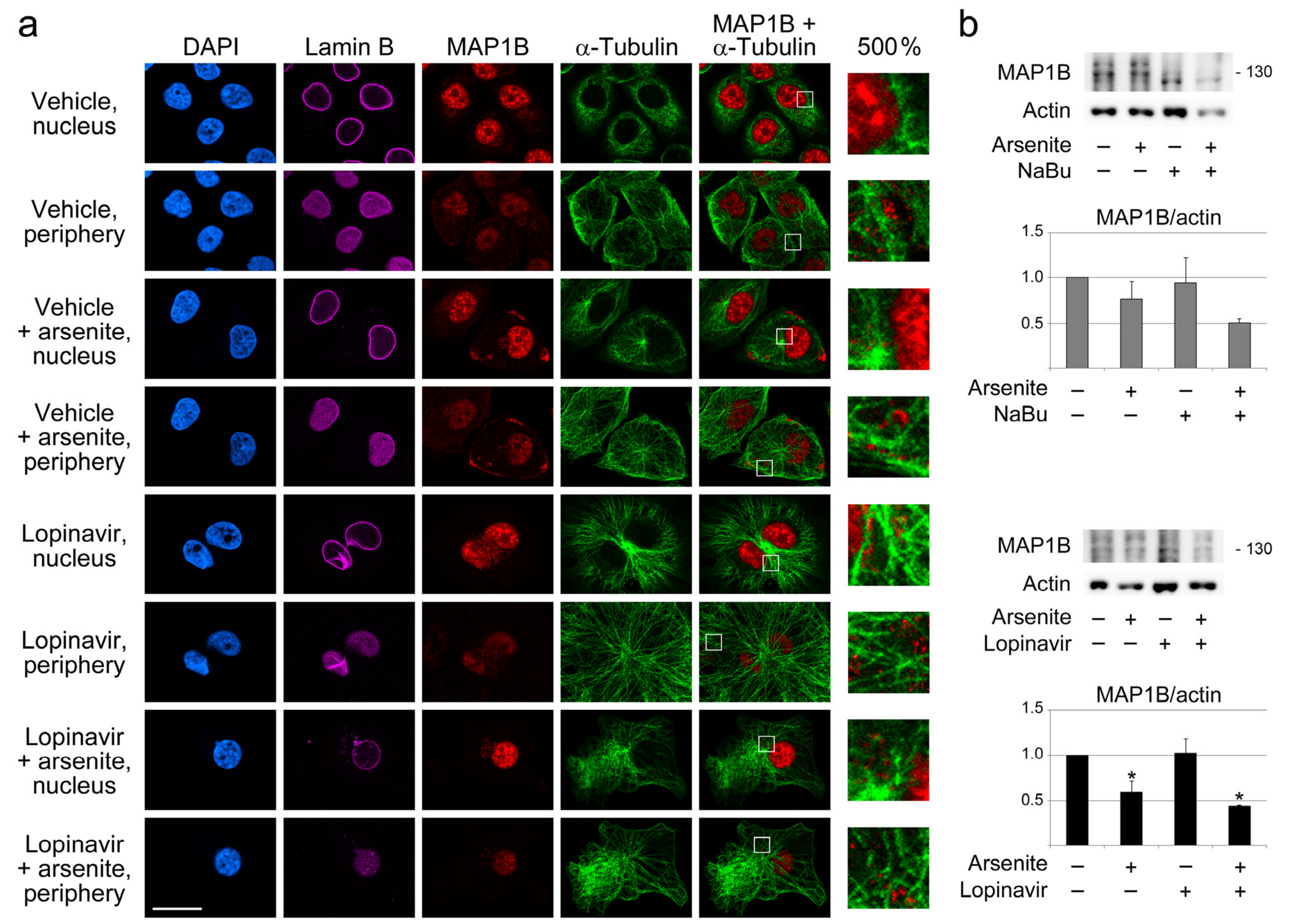

Supplementary Figure 7. Effects of IEC senescence and arsenite stress on MAP1B subcellular distribution and abundance. IECs were incubated with senescence inducers and arsenite as described in Materials and Methods. (a) Immunolocalization of MAP1B. The same cells were imaged by focusing on the nucleus or the cell periphery. Lamin B and DAPI demarcated the nuclei. Selected regions (white squares) were magnified to 500%. Size bar is 20 µm. (b) Western blots were performed for three to five independent experiments. The molecular mass of marker proteins is depicted at the right margin in kDa. Results are shown as average +SEM. One-way ANOVA combined with Bonferroni correction identified significant differences; *, $p<0.05$.

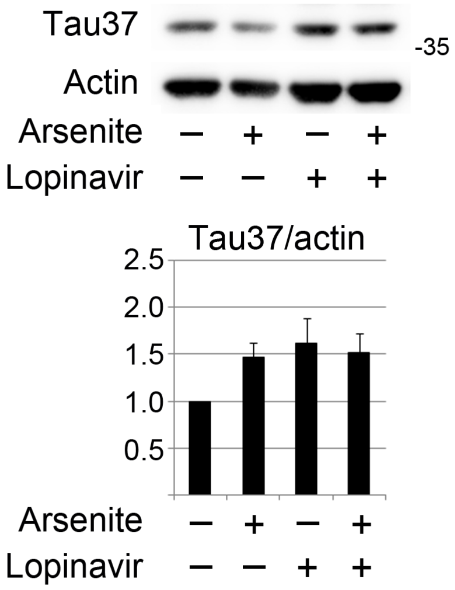

Supplementary Figure 8. Lopinavir increases the abundance of tau proteins in renal proximal epithelial cells. LLC-PK1 renal proximal tubule cells were incubated with lopinavir without or with arsenite. Crude extracts were assessed for changes in tau protein levels. The molecular mass of a marker protein is shown in kDa at the right margin. The graph depicts results for three independent experiments. Bars represent averages +SEM. One-way ANOVA combined with Bonferroni posthoc analysis did not identify significant differences of the abundance of the 37 kDa tau protein between experimental conditions.

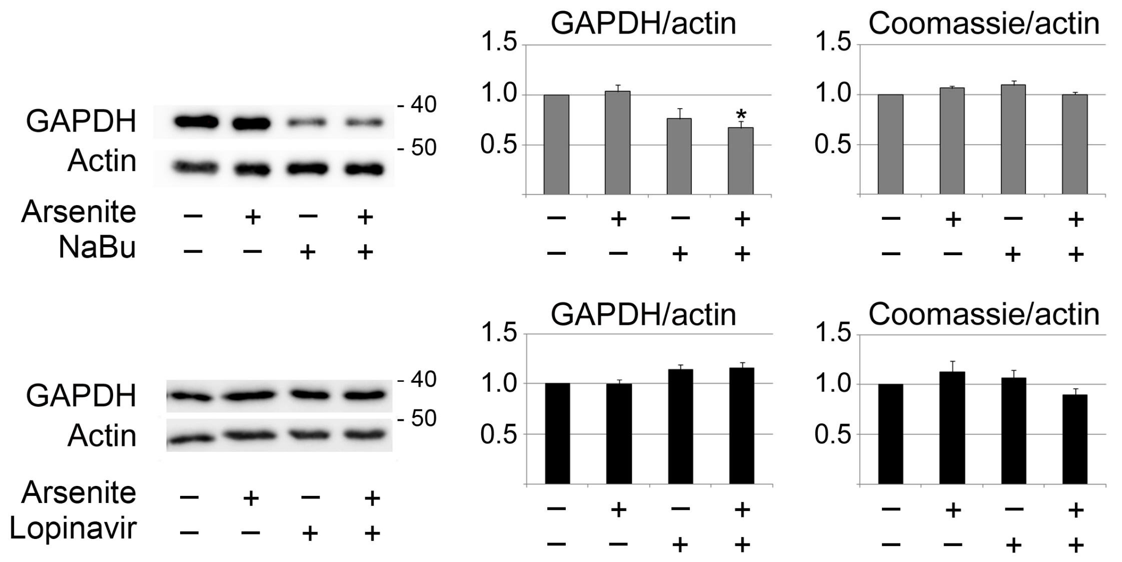

Supplementary Figure 9. Selection of actin as the appropriate loading control for Western blotting. IECs were treated with sodium butyrate (NaBu) or lopinavir and processed for Western blotting. Coomassie staining and Western blotting was performed as described in Materials and Methods section. Molecular masses of marker proteins (in kDa) are depicted at the right margin of the blots.

GAPDH/actin ratios were determined for three to six independent experiments. Coomassie/actin signals were assessed for two to three independent experiments. Each bar shows the average +SEM.

Sodium butyrate treatment reduced GAPDH abundance relative to actin; *, p<0.05 as determined by One-way ANOVA and Bonferroni post-hoc test. By contrast, there were no significant differences between the colorimetric signals for Coomassie and actin abundance.

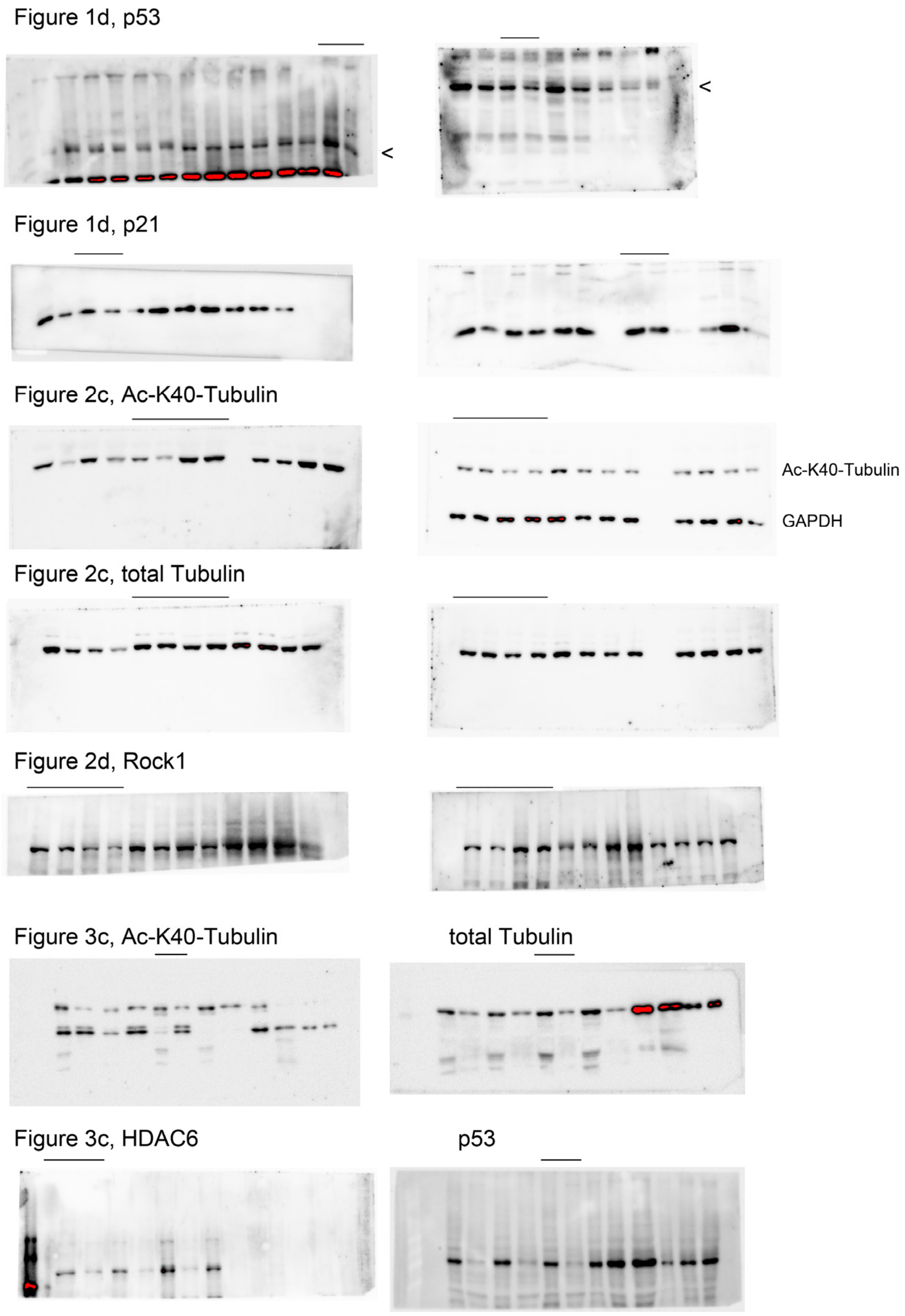

Supplementary Figure 10. Unprocessed Western blots. The original scans of the most important Western blots for figures 1 to 3 are shown. The labeling of original scans refers to the figure numbers in the text. Horizontal lines on top of the original scans mark the position of lanes that are relevant to the figures in the main text. Arrowheads mark the position of p53.

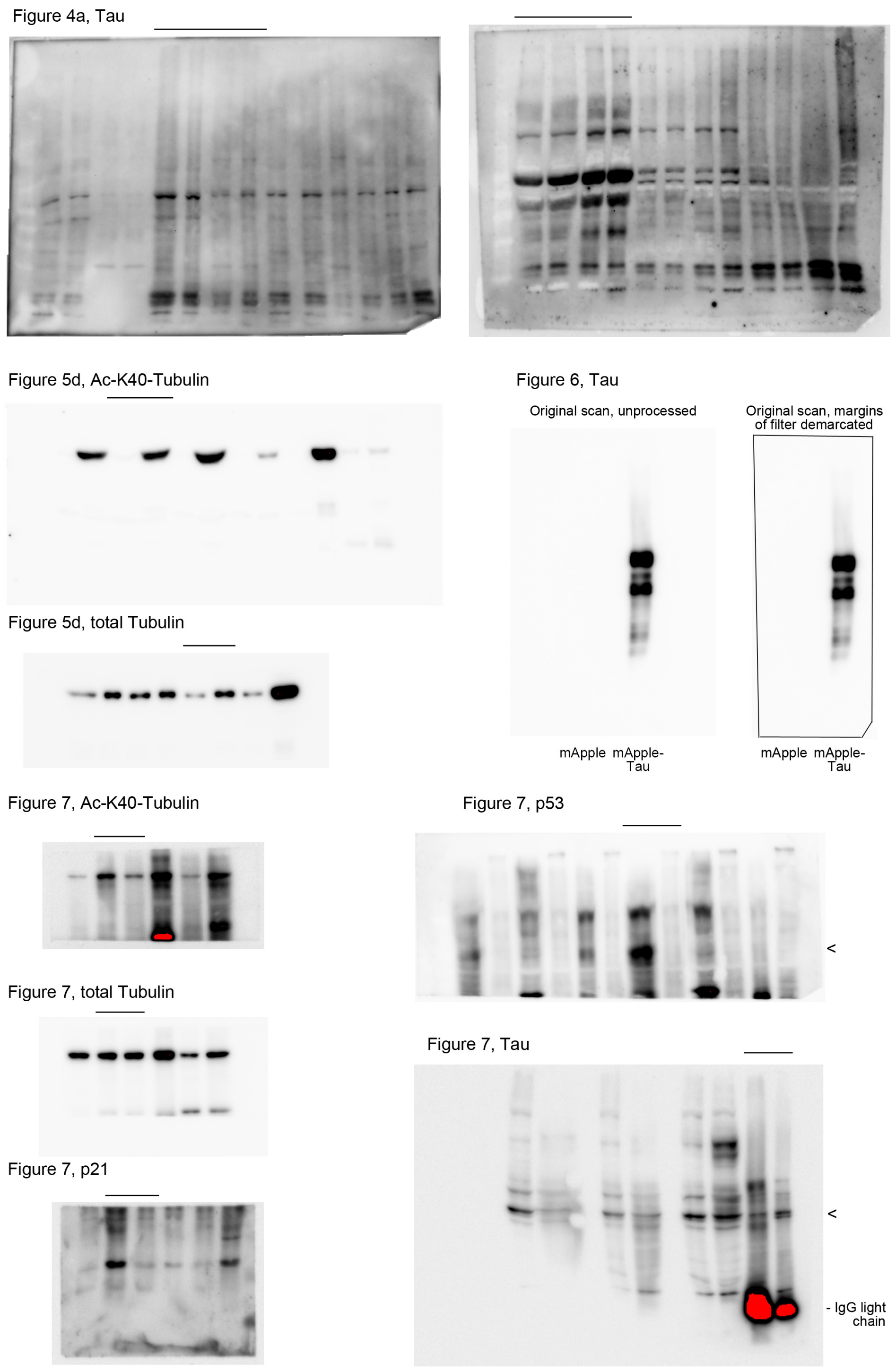

Supplementary Figure 11. Unprocessed Western blots. The original scans of the most important Western blots are depicted for figures 4 to 7. The labeling of original scans refers to the figure numbers in the text. For the original scan of the Western blot related to Fig. 6, the margins of the filter are delimited on the right side. Note that for the detection of tau in Fig. 7, HRP-conjugated secondary antibodies were bound specifically to IgG light chains. The ECL signal for the IgG light chains was saturated for this blot. Horizontal lines on top of the original scans mark the position of lanes that are relevant to the figures in the main text. For Figure 7, arrowheads mark the position of p53 and Tau.

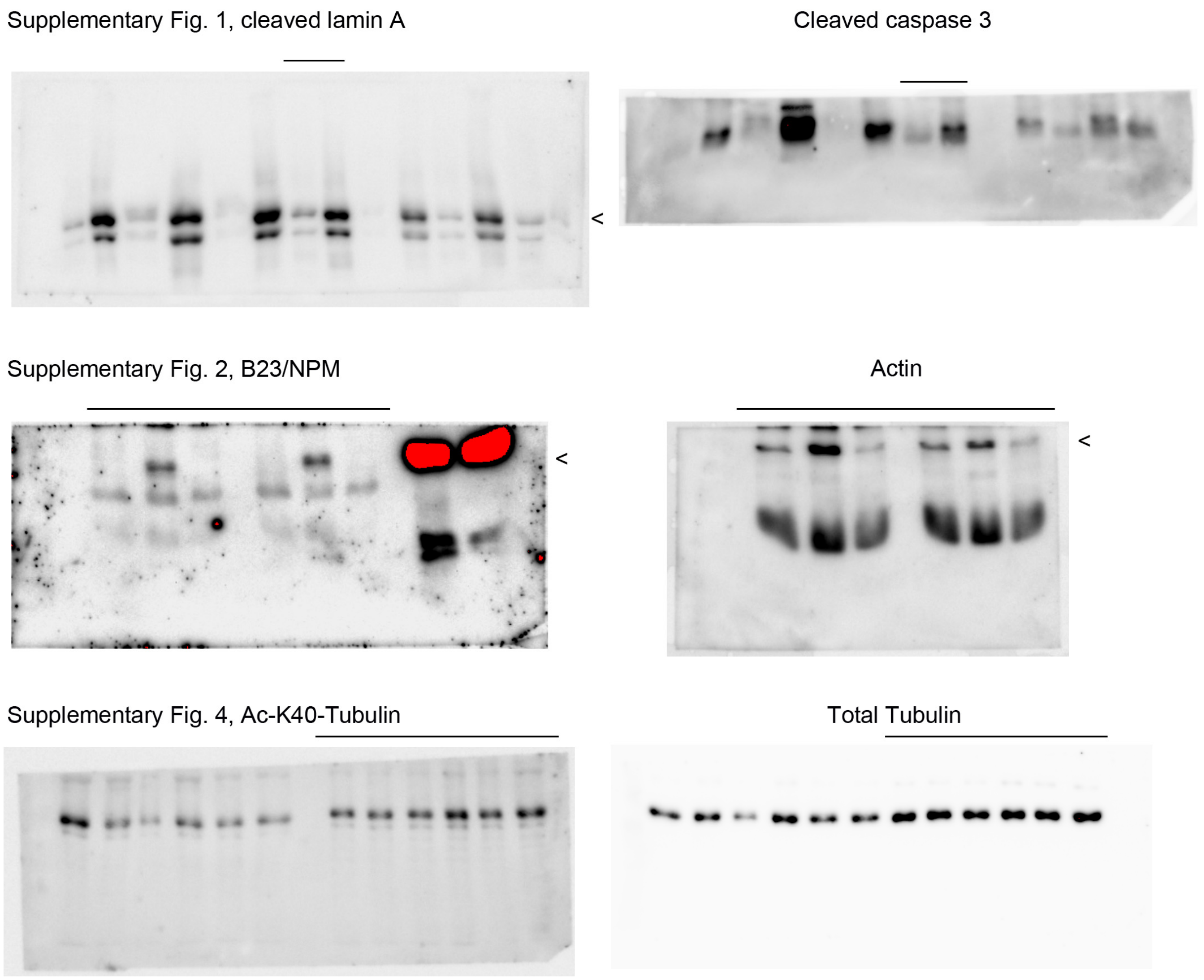

Supplementary Figure 12. Unprocessed Western blots. The original scans of the most important Western blots are shown for Supplementary figures 1 to 4. The labeling of original scans refers to the figure numbers in the Supplementary file. Horizontal lines on top of the original scans mark the position of lanes that are relevant to the figures in the Supplementary file. Arrowheads indicate the position of the bands for cleaved lamin A, B23/NPM, and actin.

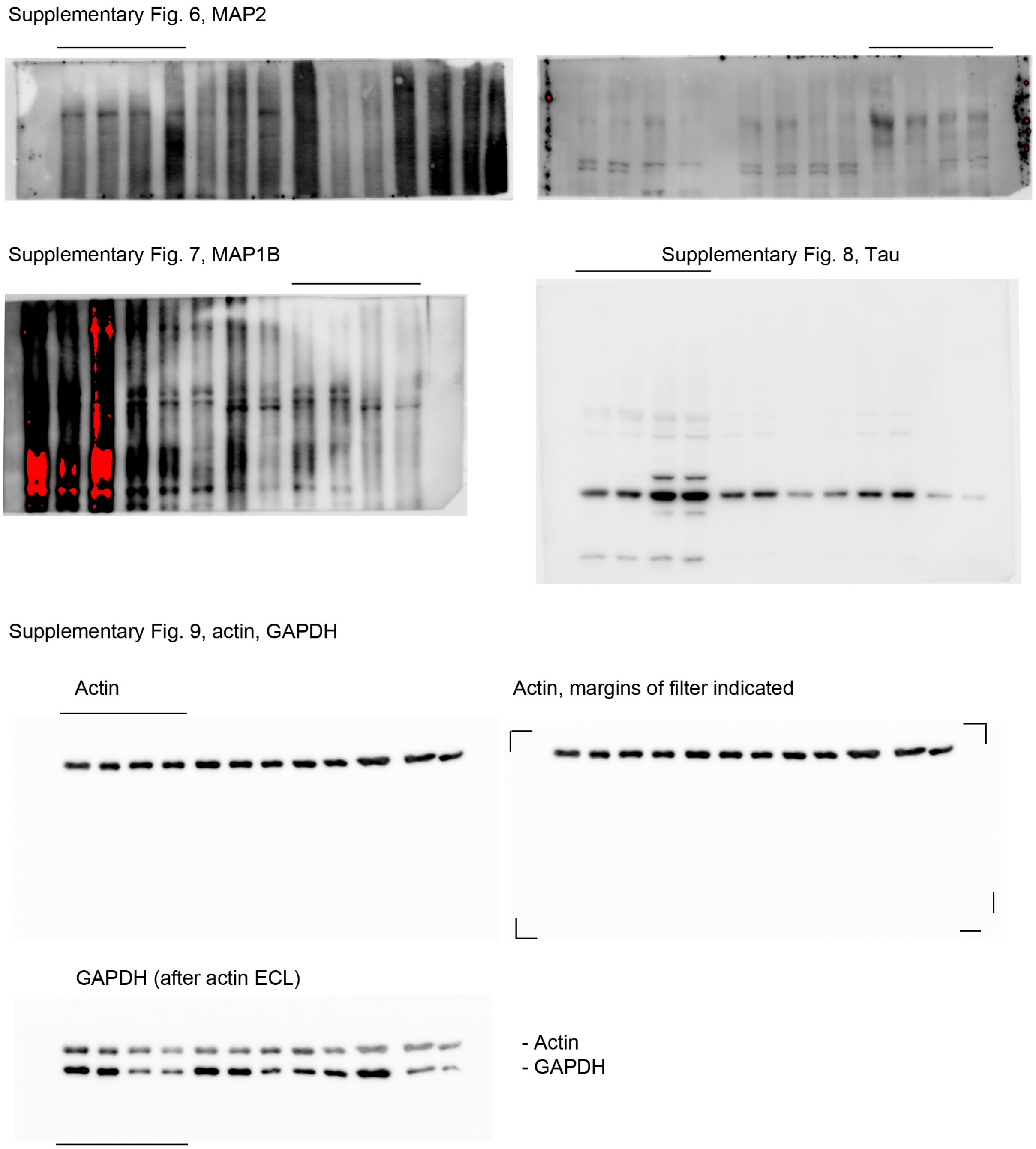

Supplementary Figure 13. Unprocessed Western blots. The original scans of the most important Western blots for Supplementary figures 6 to 9 are shown. The labeling of original scans refers to the figure numbers in the Supplementary file. Horizontal lines at the top or bottom of the original scans mark the position of lanes that are relevant to the figures in the Supplementary file.
